# Should oncological cases of upper urinary system be excluded at the beginning of the laparoscopic learning curve?

**DOI:** 10.1590/S1677-5538.IBJU.2014.0134

**Published:** 2015

**Authors:** Özgür Haki Yüksel, Alper Ötünçtemur, Emin Özbek, Fatih Uruç, Ayhan Verit

**Affiliations:** 1 Fatih Sultan Mehmet Research & Training Hospital, Dept. of Urology, Istanbul, Turkey;; 2 Okmeydani Research & Training Hospital, Dept. of Urology, Istanbul, Turkey

**Keywords:** Laparoscopy, Learning Curve, Urogenital System, complications [Subheading]

## Abstract

**Purpose:**

The place of oncological cases of upper urinary system in the laparoscopic learning curve was investigated.

**Materials and Methods:**

A total of 139 patients from two different centers underwent laparoscopic operations and were included in this retrospective study.

**Results:**

Mean operative times for oncological, and non-oncological cases were 101.3 (range 60-450), and 102.7 (45-490) minutes respectively. Fourty-two (31.3 %) patients were oncological cases. In 4 oncological cases, the surgeons switched to open surgery because of massive bleeding and six (14.2 %) oncological cases required blood transfusions during peri/postoperative periods. Pulmonary embolism was observed in one oncological case. In one non-oncological case, the surgeon switched to open surgery because of intestinal perforation and 10 (9.7 %) non-oncological cases needed blood transfusions during peri/postoperative periods. In addition, some complications such as intestinal perforation (n=1), mechanical ileus (n=1), and pulmonary embolism (n=1) were observed during postoperative period. Intestinal perforation was repaired using laparoscopic (n=1) method. Mechanical ileus was approached with open surgical technique. Mean hospital stay of the patients in the oncological and non-oncological series were 4.5 (3-23) and 4.5 (3-30) days respectively.

**Conclusion:**

We think that renal oncological cases should be included in the spectrum of laparoscopic indications even at the beginning of the learning curve. Certainly, we still share the opinion that cancer cases which require highly challenging surgeries like radical cystectomy, and prostatectomy should be postponed till to gaining of higher level of experience.

## INTRODUCTION

The popularity of laparoscopic surgeries increased because of its advantages of shorter hospital stay, rapid return to work, better cosmetic results and treatment efficacy similar to open surgery. Laparoscopy in urology was firstly used in laparoscopic pelvic lymph node dissection performed on patients with prostate cancer (1). Its first use in renal surgery was credited to Clayman et al. in 1990 who realized laparoscopic nephrectomy in a case with a renal oncocytoma measuring 3 cm in diameter (2). One year later Ehrlich et al. performed the first pediatric laparoscopic nephrectomy (3). Later on, laparoscopic surgery has been applied with increasing frequency for a larger spectrum of indications. Transperitoneal, and retroperitoneal laparoscopic ureterolithotomy operations were performed by Lipsky (1993), and Gaur (1994), respectively (4, 5). Laparoscopic pyeloplasty was realized firstly by Schuessler et al. in 1993 as a minimally invasive alternative, and it has been used with success rates competing with those of open pyeloplasty (6). Laparoscopic adrenalectomy was initially applied in 1992 by Gagner et al. (7, 8). Thanks to developments in imaging modalities, and popularization of minimally invasive interventions, laparoscopic intervention for renal cysts has taken its place among treatment modalities (9). The greatest disadvantages of laparoscopic surgery which has been introduced with a dazzling speed into urology practice for the last 20 years consist of its higher cost, and longer learning curve. In particular, reconstructive and oncological surgeries require a longer learning curve and higher operative complication rates related with laparoscopic surgeries had been seen at this period.

In our study, oncological and nononcologic cases of upper urinary system at the initial of the learning curve have been comparatively investigated.

## MATERIALS AND METHODS

In our clinics, the initial series of a total of 139 patients from two different centers who underwent laparoscopic operations between April 2010, and December 2013 were investigated in a retrospective design. The patients were operated due to renal pelvic mass (n=1), ureteral mass (n=1), renal mass (n=40), nonfunctional kidney (n=64), ureteral stone (n=10), renal stone (n=1), ureteropelvic junction obstruction (n=8), and renal cyst (n=14). All laparoscopic interventions were realized via transabdominal method in two centers by a single surgeon. In this study surgeons (n: two-single surgeon in each center) had certificate of laparoscopic training in live swine model and a limited number of human cases experiences.

### Statistical analysis

For statistical analysis, NCSS (Number Cruncher Statistical System) 2007&PASS (Power Analysis and Sample Size) 2008 Statistical Software (Utah, USA) programs were used. Study data were evaluated using descriptive statistical methods (mean, standard deviation, minimum, maximum, median, frequency, and ratio). In the intergroup comparisons of quantitative data, for parameters demonstrating normal distribution, t test and for parameters demonstrating non-parametric distribution Mann Whitney U test were used. In the evaluation of categorical variables, Pearson Chi-Square and Fisher's Exact test were used. Statistical significance was rated at p<0.01 and p<0.05 respectively.

### Operative technique

All laparoscopic interventions were performed transperitoneally after access through three ports with the patient in the 70° lateral decubitus position.

Laparoscopic renal cystectomy was started after medialization of the ipsilateral colonic segment. Gerota's fascia was opened, and the cyst was dissected from its peripheral adjacent anatomical structures. First of all, the contents of the cyst were aspirated. Then the cyst was opened, and its wall was excised 2-3 cm away from its normal parenchymal contours, and the cyst was extracted using laparoscopic scissors. The surgical wound defect was closed with perirenal adipose tissue, and Surgicel®.

In transperitoneal laparoscopic ureterolithotomy, after medialization of the colonic segment, ureter was found at the level of lower pole of the kidney, and over the psoas muscle, and suspended with a Penrose drain. Then the stone was localized, and ureter was opened vertically using a laparoscopic scissors. The stone was extracted with the aid of a right angle stone forceps, and taken away through the port. After implantation of a DJ stent, ureteral incision was closed with 4/0 absorbable monofilament sutures.

Simple and radical laparoscopic nephrectomies were performed similarly. In the right nephrectomy, after placement of ports, white line of Toldt, and triangular hepatic ligament were resected. Colonic segment over posterior hepatic ligament was mobilized, and the kidney was dissected away up to the lower renal pole, and medialized. Then the dissection was performed from medial to the superior part of the kidney to reach the renal pedicle. Firstly renal artery, and then the renal vein were clipped, and transected. In left nephrectomies, as a different approach, splenocolic ligament was released from its attachments and the colonic segment was completely medialized.

For laparoscopic nephroureterectomy, 4 ports were prepared for access sites. In addition to the surgical phases of classical nephroureterectomy, after controlling renal pedicle, ureteral track was followed, and released from its attachments down to ureteovesical junction. Then, laparoscopic complete resection technique was used to extract ureter, and its vesical cuff.

The surgical paths taken in laparoscopic partial nephrectomy was similar to laparoscopic nephrectomy but with off clamp technique.

In laparoscopic dismembered pyeloplasty, after medialization of colon, and identification of the ureteropelvic junction at the level of lower renal pole, and over psoas muscle, the ureter was spatulated without detaching from the kidney. Anastomosis was performed using 4-0 absorbable monofilament sutures.

## RESULTS

Mean age of patients was 53.5 (range 17-82). Patients' age, body mass index (BMI) and gender are shown in Table-1. Mean operative time for all operations was 102.4 (45-490) minutes. Mean operative times for oncological, and non-oncological cases were 101.3 (range 60-450) and 102.7 (45-490) minutes respectively. Forty-two (31.3 %) patients were oncological cases that underwent nephroureterectomy (n=3), partial nephrectomy (n=5), and radical nephrectomy (n=34). Laparoscopic partial nephectomies were preferred for exophitic small masses under 3 cm and performed using a non-ischemic (off-clamp) technique (Table-2).

**Table 1:** Baseline characteristics of the patients.

Variable	Oncological and reconstructive groups (n=42)	Non-oncological group (n=97)	P
Age (years)(mean/range)	56/30-82	52.4/17-75	0.0031
BMI (kg/m^2^)(mean±SD)	24.1±3.1	24.5±3.3	0.5052
Gender (male/female)	25/17	57/40	0.933

**Table 2:** Renal Nephrometry score of the patients.

Variable	Partial nephrectomy group (n=5)	Radical nephrectomy group (n=34)
Low complexity	5	0
Moderate complexity	0	20
High complexity	0	14

Mean hospital stay of the oncological cases was 4, 5 (3-23) days. In 4 oncological cases, the surgeons switched to open surgery due to massive bleeding or inability to progress. Postoperative hospital stay of one patient prolonged because of postoperative respiratory distress due to probable pulmonary embolism that resulted in minimal pleural effusion treated with medical therapy. Six (14.2 %) oncological cases required blood transfusion during peri/postoperative periods.

Mean hospital stay of the non-oncological cases was 4, 5 (3-30) days. Postoperative hospital stay of one patient prolonged because of postoperative respiratory distress due to probable pulmonary embolism with minimal pleural effusion.

In one oncological case, the surgeons switched to open surgery due to intestinal perforation. Ten (9.7%) non-oncological cases required blood transfusion during peri/postoperative periods. During postoperative period, intestinal perforation (n=1), mechanical ileus (n=1), and pulmonary embolism (n=1) were observed. Intestinal perforation was repaired using laparoscopic (n=1) methods. While mechanical ileus was treated with open surgical technique, pulmonary embolism was approached with medical therapy.

Complications of both groups are summarized in Table-3. In our study; peri/postoperative complications were evaluated separately and together. Postoperative complications classification was made according to the Clavien-Dindo grading system (10). However statistical evaluation considered the total number of complications due to limited number of cases in the groups.

**Table 3:** Perioperative data and complications of the patients.

Variable		Oncological group (n=42)	Non-oncological group (n=97)	P Value
**Operative time (min)**				
	Mean	101.3	102.7	0.7101
	Range	60-450	45-490	
**Hospital stay (d)**				
	Mean	4.5	4.5	1.0000
	Range	3-23	3-30	
**Intraoperative complications (n)**				
	Massive bleeding	4(%9.5)	0	0.008
	Open conversion	4 (%9.5)	1(%1)	0.029
	Intestinal perforation	0	1(%1)	1.000
	Overall	4 (%9.5)	1(%1)	0.008
**Postoperative complication grade Clavien-Dindo grading system (n)**				
	I	35	83	
	II	3(%7.9)	11(%11.5)	0.756
	IIIa			
	IIIb	0	2(%2.1)	1.000
Overall Complications		7(%16.6)	14(%14.4)	**0.515**

The patients were mobilized within an average of 1.1 (1–2) days. During the postoperative period, non-steroidal anti-inflammatory drugs were used for analgesia. Urethral catheters, and drains of the patients were removed at an average of 2 (1–3) and 3.3 (2–7) days, respectively.

The parameters such as the time to mobilization, duration of non-steroidal anti-inflammatory drug use, catheterization and drain removal were not different when compared with oncological series.

## DISCUSSION

In recent years, with the development of laparoscopic instruments, devices, and technology, laparoscopy has taken an important place in the management of genitourinary problems. Generally, the duration of postoperative rehabilitation process is considerably shorter following laparoscopic surgeries. Besides, the need for analgesia decreases and the patients' recovery are better. Thus the hospital stay is shortened and in addition, patients are able return faster to routine daily activities.

The advantages of transperitoneal approaches include a larger surgical field, easy identification of distinct anatomical boundaries of spleen, liver, and colon, and improved maneuvering capability. Generally retroperitoneal approaches ensure shorter hospital stay, and lower complication rates despite of the difficulty of working within a limited narrow surgical field. Besides, retroperitoneal interventions can be applied easily in cases which had previously undergone intra-abdominal surgery, and renal pedicle can be brought under control at an earlier stage of the operation. Our two centers preferred transperitoneal approach for all patients.

It is quite evident that operative time is not the main determinative criterion for the learning curve. However, operative times shorten or even remain stable after an increase in the number of cases (11). In their series of 100 cases with laparoscopic nephrectomies, Gill et al. estimated mean operative times as 175, and 163 minutes for the first, and the next 50 cases of their series (12). Kanno et al. reported decrease in intraoperative complication rates as the operative times shorten (13). Mean operative time of all interventions was 102.4 (range 45-490) minutes (for oncological, and non-oncological cases 101.3 (range 60-450), and 102.7 (45-490) minutes, respectively).

Blood loss might be an important determinative factor for the learning curve. However, in only large-scale studies blood loss creates a difference. In our series the need for transfusion was detected in 14.2, and 9.7 % in oncological, and non-oncological series respectively.

Number of cases to be performed to develop competence for laparoscopic surgery is debatable. Phillips et al. detected a decrease in operative times after the first 20 cases (14). Vallancien et al. in their large-scale series consisting of 1311 cases which included various types of surgery (ie. nephrectomy, cystectomy, prostatectomy etc.) reported different levels of experience dependent on the type, and difficulty levels of surgery, and also indicated at least 30 cases should be performed using laparoscopic methods to gain adequate competence for the first learning curve (15).

Gill et al. demonstrated a dramatic drop from 34 to 4 % in complication rates after being competent in the laparoscopic learning curve (16, 17). However in another study, any significant difference was not detected between rates of complication, transfusion, conversion to open surgery, and amount of blood loss with accumulating experience. However, a difference between the novice and experienced groups was detected as for the amount of blood loss, and the need for transfusion (10). In a study where 2775 laparoscopic interventions were performed within 12 years at the John Hopkins Hospital, relatively higher (22.2 %) complication rates were observed (18).

In a series of 150 patients who had all undergone transperitoneal laparoscopic surgeries, mean operative time was 187.6±46.56 minutes. The patients were divided into 10 groups, and the first three groups were operated on by novice, and the remaining seven groups by experienced surgeons. Intraoperative complication rates were 13.3% in the first three groups, while it was found to be 8.6% in the remaining groups. Postoperative complication rates were 8.9%, and 9.5% in the first 3, and the next 7 groups, respectively. A significant difference was found in the amount of blood loss and transfusion rates (236.4±41.85 mL vs 191.5±21.9 mL, and 17.8% vs 4.8%, respectively). This study considered experience with 15 successive operations to be adequate to gain experience (17). Total complication rates observed in oncological and non-oncological cases, in our two-centered study which was presented as a pilot series were 16.6, and 14.4 %, respectively. The present complications – mostly due to the blood loss - seemed to be high in comparison with the current literature. The need for transfusion was 14.2, and 9.7 % in oncological, and non-oncological series respectively. With the exclusion of bleeding problems, these rates dropped to 2.3% and 4.1% in oncological and non-oncological cases; respectively. Our explanations are: firstly, partial nephrectomy cases made via off clamp technique, secondly, suturing is a well-known time consuming part for the inexperienced learners (19). As in our patients, off-clamp laparoscopic partial nephrectomy was suggested in cases with low renal nephrometry score (20, 21). The evaluation of aforementioned studies suggests that apparently a complete consensus has not been reached so far regarding laparoscopic learning curve. Though laparoscopic experience has been associated with the number of cases, and complication rates, diverse outcomes have been observed in various studies performed in different centers. Besides, a clear-cut opinion has not been formulated about the initial laparoscopic operations.

## CONCLUSIONS

In the clinical practice, the beginners of the Urologic laparoscopy have a tendency to postpone the oncologic cases. However, in the present study, we compared our initial series of laparoscopic upper urinary system oncological and non-oncological cases and couldn't observe any major differences in regard to the complication rate and main parameters such as operation time and hospital stay. Therefore, we think that renal oncological cases should be included in the spectrum of laparoscopic indications even at the very early period of the learning curve. However, we still maintain the opinion that cancer cases which require highly challenging surgeries such as radical cystectomy, and prostatectomy should be postponed till to the gaining of higher level of surgical experience.
